# Supplementary material for: Characteristics and practices of school-based cluster randomised controlled trials for improving health outcomes in pupils in the UK: a systematic review protocol
Source: BMJ Open. 2021 Feb 15;11(2):e044143. doi: 10.1136/bmjopen-2020-044143 (PMC7887361; doi:10.1136/bmjopen-2020-044143)
Supplement: Supplementary data [file bmjopen-2020-044143supp001.pdf]

Table S1: MEDLINE search strategy

MEDLINE Search strategy
Terms for Randomised Controlled trials:
1. random:.mp.
2. trial. ab,kw, ti.
Cluster design-related terms:
3. “cluster*”.ab, kw, ti.
4. “group*”.ab, kw, ti.
5. “communit*”.ab, kw, ti.
6. 3 OR 4 OR 5
School MESH term:
7. exp Schools/
Highest precision:
8. 1 AND 2 AND 6 AND 7
9. 8 limited to English language
