# Supplementary material for: Characteristics and practices of school-based cluster randomised controlled trials for improving health outcomes in pupils in the UK: a systematic review protocol
Source: BMJ Open. 2021 Feb 15;11(2):e044143. doi: 10.1136/bmjopen-2020-044143 (PMC7887361; doi:10.1136/bmjopen-2020-044143)
Supplement: Author's manuscript [file bmjopen-2020-044143.draft_revisions.pdf]

BMJ Open

BMJ Open is committed to open peer review. As part of this commitment we make the peer review history of every article we publish publicly available.

When an article is published we post the peer reviewers' comments and the authors' responses online. We also post the versions of the paper that were used during peer review. These are the versions that the peer review comments apply to.

The versions of the paper that follow are the versions that were submitted during the peer review process. They are not the versions of record or the final published versions. They should not be cited or distributed as the published version of this manuscript.

BMJ Open is an open access journal and the full, final, typeset and author-corrected version of record of the manuscript is available on our site with no access controls, subscription charges or pay-per-view fees (http://bmjopen.bmj.com).

If you have any questions on BMJ Open's open peer review process please email info.bmjopen@bmj.com

BMJ Open

Characteristics and practices of school-based cluster randomised controlled trials for improving health outcomes in pupils in the UK: a systematic review protocol

Journal:	*BMJ Open*
Manuscript ID	bmjopen-2020-044143
Article Type:	Protocol
Date Submitted by the Author:	26-Aug-2020
Complete List of Authors:	Parker, Kitty; University of Exeter, NIHR ARC South West Peninsula<br>Nunns, Michael; University of Exeter, College of Medicine and Health<br>Xiao, ZhiMin; University of Exeter, Graduate School of Education<br>Ford, Tasmin; University of Cambridge<br>Ukoumunne, Obioha; University of Exeter, NIHR ARC South West Peninsula (PenARC)
Keywords:	STATISTICS & RESEARCH METHODS, Community child health < PAEDIATRICS, PUBLIC HEALTH

SCHOLARONE™
Manuscripts

I, the Submitting Author has the right to grant and does grant on behalf of all authors of the Work (as defined in the below author licence), an exclusive licence and/or a non-exclusive licence for contributions from authors who are: i) UK Crown employees; ii) where BMJ has agreed a CC-BY licence shall apply, and/or iii) in accordance with the terms applicable for US Federal Government officers or employees acting as part of their official duties; on a worldwide, perpetual, irrevocable, royalty-free basis to BMJ Publishing Group Ltd ("BMJ") its licensees and where the relevant Journal is co-owned by BMJ to the co-owners of the Journal, to publish the Work in this journal and any other BMJ products and to exploit all rights, as set out in our licence.

The Submitting Author accepts and understands that any supply made under these terms is made by BMJ to the Submitting Author unless you are acting as an employee on behalf of your employer or a postgraduate student of an affiliated institution which is paying any applicable article publishing charge ("APC") for Open Access articles. Where the Submitting Author wishes to make the Work available on an Open Access basis (and intends to pay the relevant APC), the terms of reuse of such Open Access shall be governed by a Creative Commons licence – details of these licences and which Creative Commons licence will apply to this Work are set out in our licence referred to above.

Other than as permitted in any relevant BMJ Author's Self Archiving Policies, I confirm this Work has not been accepted for publication elsewhere, is not being considered for publication elsewhere and does not duplicate material already published. I confirm all authors consent to publication of this Work and authorise the granting of this licence.

For peer review only

Characteristics and practices of school-based cluster randomised controlled trials for improving health outcomes in pupils in the UK: a systematic review protocol

Corresponding author

Ms Kitty Parker, NIHR ARC South West Peninsula, University of Exeter, Exeter, United Kingdom. Room 2.16, South Cloisters, St Luke's Campus, 79 Heavitree Rd, Exeter, EX1 1TX Email: kp477@exeter.ac.uk Telephone: (+44) 01392 727588

Co-authors

Dr Michael Nunns, College of Medicine and Health, University of Exeter, Exeter, United Kingdom.

Dr ZhiMin Xiao, Graduate School of Education, University of Exeter, Exeter, United Kingdom.

Professor Tamsin Ford, Department of Psychiatry, University of Cambridge, Cambridge, United Kingdom.

Dr Obioha C Ukoumunne, NIHR ARC South West Peninsula, University of Exeter, Exeter, United Kingdom

Word count: 2811, excluding title page, abstract, references, figures and tables

Key words

Cluster Randomised Trials

Schools

Systematic review

For peer review only - http://bmjopen.bmj.com/site/about/guidelines.xhtml

ABSTRACT

Introduction:

Cluster randomised trials (CRTs) are studies in which groups of participants (clusters) rather than the individuals themselves are randomised to trial arms. CRTs are becoming increasingly relevant for evaluating interventions delivered in school settings for improving the health of children. Schools (clusters) are a convenient setting for health interventions targeted at children and the CRT design respects the clustered structure in schools (i.e., pupils within classrooms/teachers within schools). Some of the methodological challenges of CRTs, such as ethical considerations for enrolment of children into trials and how best to handle the analysis of data from participants (pupils) that change clusters (schools), may be more salient for the school setting. A better understanding of the characteristics and methodological considerations of school-based CRTs of health interventions would inform the design of future similar studies. To our knowledge this is the only systematic review to focus specifically on the characteristics and methodological practices of CRTs delivered in schools to evaluate interventions for improving health outcomes in pupils in the UK.

Methods and analysis:

We will search for CRTs published from inception to 30th June 2020 inclusively indexed in Ovid (MEDLINE). We will identify relevant articles through title and abstract screening, and subsequent full-text screening for eligibility against pre-defined inclusion criteria. Disagreements will be resolved through discussion. Two independent reviewers will extract data for each study using a pre-piloted data extraction form. Findings will be summarised using descriptive statistics and graphs.

Ethics and dissemination:

This methodological systematic review does not require ethical approval as only secondary data extracted from papers will be analysed and the data are not linked to individual participants. After completion of the systematic review, the data will be analysed, and the findings disseminated through peer-reviewed publications and scientific meetings. The review is registered on PROSPERO (Registration number: CRD42020201792).

For peer review only - http://bmjopen.bmj.com/site/about/guidelines.xhtml

ARTICLE SUMMARY

Strengths and limitations of this study

- To our knowledge, this is the first systematic review to describe the characteristics and methodological practices of school-based cluster randomised trials (CRTs) in the UK.
- The review has a defined search strategy that is tailored to identifying school-based CRTs, eligibility criteria, and pre-piloted screening and data extraction strategies to minimise inaccuracies.
- Two independent reviewers will perform screening and data extraction, with any uncertainty resolved by consulting a third reviewer.
- The findings of this review will inform the design of future similar studies.
- The review will focus on studies conducted in the UK in order to align with available resources and create a relevant and focused review. There is the possibility that we are missing the opportunity to learn from studies in countries that have a similar education system to the UK.

For peer review only

For peer review only - http://bmjopen.bmj.com/site/about/guidelines.xhtml

INTRODUCTION

Cluster randomised trials (CRTs), also known as group randomised or place randomised trials, are studies in which groups (clusters) of participants (e.g., general practices, organisations, areas, etc) are randomly allocated to the trial arms, rather than the individual participants on whom outcomes are measured.[1] These studies are in contrast to the more traditional individual randomised trials, where the participants themselves are randomised. The CRT design is commonly used in health care research when interventions must be delivered at the cluster level and to minimise contamination of the trial arms that might otherwise occur when individuals are randomised.[2]

A characteristic feature of cluster randomised trials is that observations on participants who are in the same cluster are usually more similar than observations on participants who are from different clusters.[2] For example, patients registered with the same general practice are more likely to have similar health outcomes than those registered with different practices.[3] This similarity, or lack of statistical independence, between observations from the same cluster means that the usual procedures for calculating sample size and analysing data in individually randomised trials should not be used in CRTs.[1] The use of standard sample size calculations are likely to result in studies that lack power to detect the specified intervention effect and the use of standard analytical methods may produce results that exaggerate evidence for the true effect of the intervention. Therefore, alternative methods should be used when conducting CRTs.

The intra-cluster correlation coefficient (ICC), denoted ρ, quantifies the similarity of observations of individuals within the same cluster. The ICC is the proportion of the total variability in the trial outcome that is between clusters (σ_b^2) as opposed to between individuals within clusters (σ_w^2):[4]

$$\rho = \frac{\sigma_b^2}{\sigma_b^2 + \sigma_w^2}$$

ρ can take values between 0 and 1. The larger ρ is, the greater the similarity between individuals within clusters, or equivalently the greater variability between clusters.

Information about ρ for the primary outcome is invaluable when designing a cluster randomised trial. It can be estimated from previous studies or pilot studies, with a similar cluster structure and outcome to the planned trial.[5 6] Authors of cluster randomised trials should report estimates of their ICCs, ideally with confidence intervals because they are

For peer review only

For peer review only - http://bmjopen.bmj.com/site/about/guidelines.xhtml

usually based on studies with relatively few clusters,[7] to aid the design of future similar studies.

When calculating the sample size in CRTs one needs to determine the total number of clusters that need to be recruited and the number of individuals that need to be recruited from within each cluster. Methods for calculating the required sample size need to take the ICC into account. When the number of participants in each cluster is fixed and known in advance, the total number of individuals required in a CRT is calculated by inflating the number of individuals that would be required in an individual randomised trial by the *design effect* (*DE*), which is function of ρ:

$$DE = 1 + (n - 1)\rho$$

where *n* is the number of participants providing outcome data in each cluster (cluster size).[8] Having calculated the total number of participants required, this is divided by the cluster size to obtain the total number of clusters that is required. For scenarios where the total number of clusters available for a trial is known, an alternative calculation based on the same approach is used to calculate the number of participants that need to be recruited from each cluster.[3]

When estimating the intervention effect in CRTs, analytical methods that take account of ρ should be used,[1] otherwise confidence intervals will be too narrow and p values will be too small, resulting in an exaggeration of the amount of evidence for a true intervention effect.[9] Furthermore, the degrees of freedom used for calculating the confidence interval and p-value for the intervention effect should take account of the number of clusters.[10,11] A distinction can be made between statistical analyses that are carried out at the cluster level and those that are carried out at the individual level.[8] For cluster-level analyses the outcome is summarised for each cluster, for example by calculating the mean for continuous outcomes or percentages for binary outcomes across individuals in the cluster. Standard analytical methods are then used to compare the outcome between the trial arms using the cluster-level summary statistics as the observations. This method of analysis is valid because the cluster is both the unit of randomisation and the unit of analysis.[2] Alternatively, analyses of individual-level data involve the application of statistical methods that allow for the within cluster correlation.[2] This approach is exemplified by methods such as mixed effects (“multilevel”) models and marginal models estimated using Generalized Estimating Equations (GEEs).

For peer review only - http://bmjopen.bmj.com/site/about/guidelines.xhtml

CRTs are increasingly used to evaluate interventions for improving health outcomes in children.[12 13] Because of the amount of time children spend in school, it provides a natural setting in which interventions for preventing health problems can delivered, participating children can be recruited, and health outcomes measured.[13] At a policy level there is increasing awareness of the potential for using the school setting to deliver, non-pharmacological, complex, prevention public health interventions.[12 14 15] Cluster randomisation is a more natural approach than individual randomisation in the school-based setting. It is often difficult to randomise individuals as pupils belong to predefined clusters (e.g., class, year group, school), and contamination between the trial arms can result as pupils interact within clusters. The CRT design respects the clustered structure in schools.

School-based cluster randomised trials share the same challenges of trials where other types of cluster are randomised. Within-cluster correlation is expected in school-based cluster randomised trials for a number of reasons: parents choose the schools their children attend and this may be related to factors associated with pupil outcomes; the school environment and culture will have a common influence on the pupils; pupils interact within schools and this can result in similar behaviours and outcomes.[12 13 16] Other recognised challenges may be even more salient for trials that randomise schools. There are additional ethical considerations for enrolment of children into trials to ensure pupils remain protected as research subjects.[12] For trials that have a long follow-up duration one needs to consider how best to handle the analysis of data from pupils that change schools (clusters) during the course of the study.[17]

Several books have been published regarding CRT methodology.[1 2 18-20] In addition there have been a number of reviews of the conduct and reporting quality of cluster randomised trials.[12 21-26] One systematic review examined the characteristics and quality of reporting of CRTs worldwide involving children[12] and highlighted the specific difficulties of conducting such studies; nearly three quarters of the included studies randomised schools as the clusters. That review and our initial scoping research suggests a sharp increase in the number of these studies. No systematic review has specifically focussed on the characteristics of CRTs delivered in the school-based setting in the UK. Such a review would: provide a pool of relevant knowledge for researchers planning future similar trials in the UK and improve their quality; highlight good practices and common methodological challenges; obtain useful trial-based data on the intra-cluster (intra-school) correlation coefficient; provide relevant parameter values for simulation-based studies that use synthetic

For peer review only - http://bmjopen.bmj.com/site/about/guidelines.xhtml

data to assess the statistical properties of methods used to analyse data from school-based cluster randomised trials.

This review aims to summarise the characteristics of, and methodological practices in, school-based CRTs with pupil health outcomes in the United Kingdom. The review examines several areas, including: participant characteristics; intervention type; recruitment, sampling and allocation methods; consent and ethical approval procedures; analysis methods; and retention. The main outcome is a description of the methodological characteristics of school-based cluster randomised trials in the UK with a health outcome. Knowledge of the study characteristics and practices of researchers will greatly aid the design of cluster randomised trials in the school health setting.

METHODS

The systematic review will describe the characteristics of cluster randomised trials with health outcomes in the school setting. This section contains a description of the methodological strategy based on guidelines from the Preferred Reporting Items for Systematic Review and Meta-Analysis (PRISMA) statement.[27] The review is registered on PROSPERO (Registration number: CRD42020201792), an international register for systematic reviews, and any changes to the protocol will be recorded.

Search strategy

Peer reviewed articles written in English, published from inception to 30th June 2020 inclusively indexed in Ovid (MEDLINE) will be the source of data for this systematic review. The search strategy was developed following an initial scoping of the focus area and in consultation with an information specialist. The search strategy combined free text and index terms for the concepts *study type* (CRTs) and *schools* (Table 1). The study type concept was developed based on the sensitive MEDLINE search strategy for identification of cluster randomised trials developed by Taljaard et al.[28] Cluster design-related terms, ‘cluster*’, ‘group*’ and ‘communit*’ were combined with the terms ‘random’ and ‘trial’, along with ‘Schools’. The search was then limited to English language as our resources make it unfeasible to translate papers.

For peer review only

Table 1: Search strategy implemented in this systematic review

Search strategy
Terms for Randomised Controlled trials:
1. random:.mp.
2. trial. Ab,kw, ti.
Cluster design-related terms:
3. "cluster*".ab, kw, ti.
4. "group*".ab, kw, ti.
5. "communit*".ab, kw, ti.
6. 3 OR 4 OR 5
School MESH term:
7. exp Schools/
Highest precision:
8. 1 AND 2 AND 6 AND 7
9. 8 limited to English language

Eligibility criteria

Eligible papers will be those reporting the results from school-based cluster randomised trials of health-related interventions in the UK for which there is a primary health outcome (including physical and mental health interventions, health attitudes and well-being) measured on pupils.

The review will include participants who are children of school age in education in the UK. Participants are pupils in pre-school, primary school and secondary school settings. The types of eligible clusters include schools themselves, year groups, classrooms, teachers or any other relevant school-related unit. Any health-related intervention(s) will be considered. There must be a control/usual care comparison group within the published article. The primary outcome must be health related and measured on pupils.

All types of cluster randomised trial design are eligible, including parallel group, crossover and stepped wedge trials. Only definitive cluster randomised trials will be included. Only studies published in English will be included and we anticipate that all studies carried out in the UK will be published in English. If more than one publication of the primary outcome result for an eligible CRT is identified, a key study report (index paper) will be designated and used for data extraction.

Papers which do not report the main findings (primary outcome) will be excluded along with pilot/ feasibility studies, protocol/design articles, process evaluations, economic evaluations/cost-effectiveness studies, statistical analysis plans, commentaries and papers

For peer review only - http://bmjopen.bmj.com/site/about/guidelines.xhtml

reporting only findings from mediation/mechanism analyses. Studies for which the primary outcome is not health based (e.g., education attainment) will be excluded.

Screening and selection

All potentially eligible studies will undergo a two-stage screening process.

Stage 1: The titles and abstracts of the studies will be retrieved from Ovid (MEDLINE) and downloaded into Endnote (X9).[29] Any duplicate citations will be removed and remaining citations will be dual screened (KP & OU) for eligibility against the inclusion criteria above. Disagreements will be resolved through discussion.

Stage 2: Full text articles will be obtained for all papers that are potentially eligible following title and abstract screening. The reviewers (KP & OU) will evaluate articles based on the inclusion criteria using a pre-piloted coding method. Any discrepancies which cannot be resolved through discussion will be sent to a third reviewer (ZMX) for a decision.

Reviewers will keep a record of any studies excluded at each step. Results will be reported using a PRISMA flow diagram.

Risk of Bias (quality) assessment

A risk of bias assessment is not necessary for this methodological review as we are not interested in the specific estimates of intervention effect in the included studies. We seek only to describe characteristics of the studies. Some of the information extracted from the papers is indicative of quality in cluster randomised trials and this will be summarised as part of our review.

Data extraction

A data extraction form will be piloted on a random sample of 10 included papers. Any modifications to the form will be made following the pilot. KP will extract data from all eligible papers, while OU will check extraction. Any uncertainty will be resolved by consulting a third reviewer (ZMX). Information will be recorded using data extraction form designed on Microsoft Excel.

For peer review only - http://bmjopen.bmj.com/site/about/guidelines.xhtml

The following data will be extracted from included articles: characteristics of the participating schools and pupils; intervention type and mode of delivery; health condition/aspect targeted by the intervention; justification for using cluster trial design; unit of randomisation (i.e., type of cluster); school-level (or other cluster-level) characteristics used to balance the randomisation; allocation ratio; length of follow-up; number of follow-ups; target sample size (i.e., number of schools and pupils); assumptions underlying sample size (e.g., intra-cluster correlation coefficient, loss to follow-up); committee that provided ethical approval; activities covered by the consent agreements; primary outcome; reporter of primary outcome (e.g., teacher, parent, pupil); method of data collection; achieved sample size; number of schools (clusters) and pupils that were lost to follow-up; analysis method used to estimate intervention effect; baseline factors that were adjusted for in the analysis; value of the intra-cluster correlation coefficient in the primary analysis model; methodological challenges that were highlighted by the authors.

Missing information that is not available in the included papers will be obtained from corresponding protocol papers and other sibling publications for the studies. Authors will be contacted for missing or incomplete information and given 2 weeks to respond.

Data analysis

No formal sample size in terms of the number of required eligible papers has been calculated because we are seeking to obtain all published school-based cluster randomised trials in the United Kingdom to date published in Ovid (MEDLINE). Meta-analysis is not appropriate as the review is focused on summarising methodological characteristics. Study characteristics will be summarised using means and standard deviations (or median and interquartile ranges) for continuous variables, and numbers and percentages for categorical variables. Appropriate graphs (e.g., histogram, line graph scatterplots) will also be used to summarise specific features of the data. Challenges reported by authors will be summarised narratively.[30] Statistical analysis will be performed using Stata 16.[31]

Patient and Public Involvement

No patient involved. This is a systematic review of published studies.

For peer review only - http://bmjopen.bmj.com/site/about/guidelines.xhtml

DISCUSSION

To our knowledge, this is the first systematic review to describe the characteristics and methodological practices of school-based cluster randomised trials in the UK. We have a defined search strategy that is tailored to identifying school-based CRTs, selection criteria and a pre-piloted extraction strategy. Pilot testing, and subsequent screening and data extraction will be conducted by two independent reviewers, with disagreements resolved by consulting a third reviewer. In doing this we hope to minimise inaccuracy. Additionally, the review aims to cover a range of CRTs conducted in schools for a variety of different health conditions/areas.

We have conducted extensive scoping searches in order to best identify the studies of interest. A limitation of the review is that we will limit our search to Ovid (MEDLINE) database, thus potentially missing out on articles published in other journals (e.g. mental health interventions published in Psych Info). We have also chosen not to examine grey literature therefore potentially missing out on studies with greater methodological challenges. Also there will be no forward and backward citation searching, but we do have a clearly defined population of papers. Pilot/feasibility studies have been excluded, but there are different learning issues from such studies that will be the subject of a separate review. These decisions have been taken to enable the review to be more focused and time-effective.

Another limitation of this review is the difficulty in identifying CRTs as many papers do not explicitly use the word ‘cluster’ in the title or abstract. Therefore, we have included terms in our search such as ‘group randomised’ and ‘community randomised’ to try and improve the sensitivity, thus widening the search so not to miss any eligible papers. We have also used the exploded MESH term, ‘exp School/’, in the hope of identifying publications that may state schools or classes as their unit of randomisation.

A pragmatic decision has been made to focus on UK studies in order to align with available resources and create a relevant and focused review. There is the possibility that we are missing out on the opportunity to learn from studies in countries that have a similar education system to the UK.

Because of the amount of time children spend in school, it provides a natural setting in which interventions for preventing health problems and improving health outcomes in children can be delivered and evaluated.[13] Cluster randomised controlled trials in the school-based setting are particularly relevant for non-pharmacological interventions, such as social programmes aimed at improving public health[13] and the use of this study design is

For peer review only

increasing.[12] Through summarising the methodological aspects of health-related cluster randomised controlled trials conducted in a school-based setting, this review will provide methodology-related knowledge specific to these trials which will help researchers plan future similar studies effectively in the UK.

Ethics and dissemination: This methodological systematic review does not require ethical approval as only secondary data extracted from papers will be analysed and the data are not linked to individual participants. After completion of the systematic review, the data will be analysed, and the findings disseminated through peer-reviewed publications and scientific meetings.

Data statement: This is a systematic review of published studies. No additional data will be available beyond that which will be included in the report of the systematic review findings.

Author contributions: KP and OU conceptualised the study. KP drafted the manuscript and incorporated comments from authors for successive drafts. MN, ZMX, TF and OU contributed in design and content. All authors read and approved final manuscript. KP is the guarantor of the review.

Funding statement: This work was supported by the National Institute for Health Research (NIHR) Applied Research Collaboration South West Peninsula (NIHR ARC South West Peninsula). Our funded group National Institute for Health Research Applied Research Collaboration South West Peninsula does not have a grant number because we are part of the NIHR infrastructure. The views expressed are those of the author(s) and not necessarily those of the NHS, the NIHR or the Department of Health and Social Care.

Acknowledgments: Kitty Parker was supported by a PhD studentship funded by the National Institute for Health Research Applied Research Collaboration South West Peninsula (NIHR ARC South West Peninsula). Obioha Ukoumunne was supported by the NIHR ARC South West Peninsula. The views expressed are those of the author(s) and not necessarily those of the NHS, the NIHR or the Department of Health and Social Care.

Competing interests statement. None declared.

For peer review only

References

1. Eldridge S, Kerry S. A Practical Guide to Cluster Randomised Trials in Health Services Research. Chichester, West Sussex: John Wiley & Sons 2012.
2. Donner A, Klar N. Design and Analysis of Cluster Randomization Trials in Health Research: Wiley 2000.
3. Campbell MJ. Cluster randomized trials in general (family) practice research. *Stat Methods Med Res* 2000;9(2):81-94. doi: 10.1177/096228020000900202 [published Online First: 2000/08/18]
4. Eldridge S, Ukoumunne O, Carlin J. The intra-cluster correlation coefficient in cluster randomized trials: a review of definitions. *International Statistical Review* 2009;77:378 - 94.
5. Kerry SM, Bland JM. The intracluster correlation coefficient in cluster randomisation. *Bmj* 1998;316(7142):1455. doi: 10.1136/bmj.316.7142.1455 [published Online First: 1998/06/06]
6. Eldridge SM, Ukoumunne OC, Carlin JB. The Intra-Cluster Correlation Coefficient in Cluster Randomized Trials: A Review of Definitions. *International Statistical Review* 2009;77(3):378-94. doi: 10.1111/j.1751-5823.2009.00092.x
7. Ukoumunne OC. A comparison of confidence interval methods for the intraclass correlation coefficient in cluster randomized trials. *Stat Med* 2002;21(24):3757-74. doi: 10.1002/sim.1330 [published Online First: 2002/12/17]
8. Eldridge S, Kerry S. A Practical Guide to Cluster Randomised Trials in Health Services Research: Wiley 2012.
9. Bland JM, Kerry SM. Statistics notes. Trials randomised in clusters. *Bmj* 1997;315(7108):600. doi: 10.1136/bmj.315.7108.600 [published Online First: 1997/09/26]
10. Kahan BC, Forbes G, Ali Y, et al. Increased risk of type I errors in cluster randomised trials with small or medium numbers of clusters: a review, reanalysis, and simulation study. *Trials* 2016;17(1):438. doi: 10.1186/s13063-016-1571-2
11. Cornfield J. Randomization by group: a formal analysis. *Am J Epidemiol* 1978;108(2):100-2. doi: 10.1093/oxfordjournals.aje.a112592 [published Online First: 1978/08/01]
12. Walleser S, Hill SR, Bero LA. Characteristics and quality of reporting of cluster randomized trials in children: reporting needs improvement. *Journal of Clinical Epidemiology* 2011;64(12):1331-40. doi: https://doi.org/10.1016/j.jclinepi.2011.04.006
13. Goesling B. A Practical Guide to Cluster Randomized Trials in School Health Research. *Journal of School Health* 2019;89(11):916-25. doi: 10.1111/josh.12826
14. Crocetti MT, Amin DD, Scherer R. Assessment of risk of bias among pediatric randomized controlled trials. *Pediatrics* 2010;126(2):298-305. doi: 10.1542/peds.2009-3121 [published Online First: 2010/07/14]
15. Thomson D, Hartling L, Cohen E, et al. Controlled trials in children: quantity, methodological quality and descriptive characteristics of pediatric controlled trials published 1948-2006. *PLoS One* 2010;5(9) doi: 10.1371/journal.pone.0013106 [published Online First: 2010/10/12]
16. Shackleton N, Hale D, Bonell C, et al. Intraclass correlation values for adolescent health outcomes in secondary schools in 21 European countries. *SSM Popul Health* 2016;2:217-25. doi: 10.1016/j.ssmph.2016.03.005
17. Schweig JD, Pane JF. Intention-to-treat analysis in partially nested randomized controlled trials with real-world complexity. *International Journal of Research & Method in Education* 2016;39(3):268-86. doi: 10.1080/1743727X.2016.1170800
18. Campbell M, Walters S. How to Design, Analyse and Report Cluster Randomised Trials in Medicine and Health Related Research. Hoboken, NJ: John Wiley & Sons 2014.
19. Hayes R, Moulton L. Cluster Randomised Trials. Boca Raton, FL: Chapman and Hall/CRC Press 2009.
20. Murray D. Design and Analysis of Group-Randomized Trials. New York: Oxford University Press 1998.

For peer review only - http://bmjopen.bmj.com/site/about/guidelines.xhtml

21. Eldridge SM, Ashby D, Feder GS, et al. Lessons for cluster randomized trials in the twenty-first century: a systematic review of trials in primary care. *Clin Trials* 2004;1(1):80-90. doi: 10.1191/1740774504cn006rr [published Online First: 2005/11/12]
22. Fiero MH, Huang S, Oren E, et al. Statistical analysis and handling of missing data in cluster randomized trials: a systematic review. *Trials* 2016;17:72-72. doi: 10.1186/s13063-016-1201-z
23. Ivers NM, Taljaard M, Dixon S, et al. Impact of CONSORT extension for cluster randomised trials on quality of reporting and study methodology: review of random sample of 300 trials, 2000-8. *BMJ* 2011;343:d5886. doi: 10.1136/bmj.d5886
24. DONNER A, BROWN KS, BRASHER P. A Methodological Review of Non-Therapeutic Intervention Trials Employing Cluster Randomization, 1979–1989. *Int J Epidemiol* 1990;19(4):795-800. doi: 10.1093/ije/19.4.795
25. Simpson JM, Klar N, Donnor A. Accounting for cluster randomization: a review of primary prevention trials, 1990 through 1993. *Am J Public Health* 1995;85(10):1378-83. doi: 10.2105/ajph.85.10.1378 [published Online First: 1995/10/01]
26. Varnell SP, Murray DM, Janega JB, et al. Design and analysis of group-randomized trials: a review of recent practices. *Am J Public Health* 2004;94(3):393-9. doi: 10.2105/ajph.94.3.393 [published Online First: 2004/03/05]
27. Moher D, Liberati A, Tetzlaff J, et al. Preferred reporting items for systematic reviews and meta-analyses: the PRISMA statement. *BMJ* 2009;339:b2535. doi: 10.1136/bmj.b2535
28. Taljaard M, McGowan J, Grimshaw JM, et al. Electronic search strategies to identify reports of cluster randomized trials in MEDLINE: low precision will improve with adherence to reporting standards. *BMC Med Res Methodol* 2010;10:15-15. doi: 10.1186/1471-2288-10-15
29. EndNote [program]. EndNote X9 version. Philadelphia, PA: Clarivate, 2013.
30. Popay J, Roberts H, Sowden A, et al. Guidance on the conduct of narrative synthesis in systematic reviews: A product from the ESRC Methods Programme2006.
31. Stata16 [program]. Release 16 version. College Station, TX: StataCorp LLC, 2019.

For peer review only

For peer review only - http://bmjopen.bmj.com/site/about/guidelines.xhtml

PRISMA-P 2015 Checklist

This checklist has been adapted for use with systematic review protocol submissions to BioMed Central journals from Table 3 in Moher D et al: Preferred reporting items for systematic review and meta-analysis protocols (PRISMA-P) 2015 statement. *Systematic Reviews* 2015 **4**:1

An Editorial from the Editors-in-Chief of *Systematic Reviews* details why this checklist was adapted - **Moher D, Stewart L & Shekelle P:** Implementing PRISMA-P: recommendations for prospective authors. *Systematic Reviews* 2016 **5**:15

Section/topic	#	Checklist item	Information reported: Yes	Information reported: No	Line number(s)
**ADMINISTRATIVE INFORMATION**					
**Title**					
Identification	1a	Identify the report as a protocol of a systematic review	☒	☐	3
Update	1b	If the protocol is for an update of a previous systematic review, identify as such	☐	☒	N/A
**Registration**	2	If registered, provide the name of the registry (e.g., PROSPERO) and registration number in the Abstract	☒	☐	55
**Authors**					
Contact	3a	Provide name, institutional affiliation, and e-mail address of all protocol authors; provide physical mailing address of corresponding author	☒	☐	6-21
Contributions	3b	Describe contributions of protocol authors and identify the guarantor of the review	☒	☐	347-350
**Amendments**	4	If the protocol represents an amendment of a previously completed or published protocol, identify as such and list changes; otherwise, state plan for documenting important protocol amendments	☐	☒	N/A
**Support**					
Sources	5a	Indicate sources of financial or other support for the review	☒	☐	352-357
Sponsor	5b	Provide name for the review funder and/or sponsor	☒	☐	352-357
Role of sponsor/funder	5c	Describe roles of funder(s), sponsor(s), and/or institution(s), if any, in developing the protocol	☒	☐	359-363
**INTRODUCTION**					
**Rationale**	6	Describe the rationale for the review in the context of what is already known	☒	☐	169-182

BioMed Central
The Open Access Publisher

Section/topic	#	Checklist item	Information reported Yes	Information reported No	Line number(s)
**Objectives**	7	Provide an explicit statement of the question(s) the review will address with reference to participants, interventions, comparators, and outcomes (PICO)	☒	☐	184-191
**METHODS**					
**Eligibility criteria**	8	Specify the study characteristics (e.g., PICO, study design, setting, time frame) and report characteristics (e.g., years considered, language, publication status) to be used as criteria for eligibility for the review	☒	☐	216-237
**Information sources**	9	Describe all intended information sources (e.g., electronic databases, contact with study authors, trial registers, or other grey literature sources) with planned dates of coverage	☒	☐	203-204
**Search strategy**	10	Present draft of search strategy to be used for at least one electronic database, including planned limits, such that it could be repeated	☒	☐	202-211
STUDY RECORDS					
Data management	11a	Describe the mechanism(s) that will be used to manage records and data throughout the review	☒	☐	241-242, 263-264
Selection process	11b	State the process that will be used for selecting studies (e.g., two independent reviewers) through each phase of the review (i.e., screening, eligibility, and inclusion in meta-analysis)	☒	☐	239 – 250
Data collection process	11c	Describe planned method of extracting data from reports (e.g., piloting forms, done independently, in duplicate), any processes for obtaining and confirming data from investigators	☒	☐	259-264
**Data items**	12	List and define all variables for which data will be sought (e.g., PICO items, funding sources), any pre-planned data assumptions and simplifications	☒	☐	266-278
**Outcomes and prioritization**	13	List and define all outcomes for which data will be sought, including prioritization of main and additional outcomes, with rationale	☒	☐	185-189, 266-278
**Risk of bias in individual studies**	14	Describe anticipated methods for assessing risk of bias of individual studies, including whether this will be done at the outcome or study level, or both; state how this information will be used in data synthesis	☒	☐	252-257
DATA					
**Synthesis**	15a	Describe criteria under which study data will be quantitatively synthesized	☒	☐	283-292
	15b	If data are appropriate for quantitative synthesis, describe planned summary measures, methods of handling data, and methods of combining data from studies, including any planned exploration of consistency (e.g., I^2, Kendall's tau)	☒	☐	283-292
	15c	Describe any proposed additional analyses (e.g., sensitivity or subgroup analyses, meta-regression)	☒	☐	283-292
	15d	If quantitative synthesis is not appropriate, describe the type of summary planned	☒	☐	283-292

BioMed Central
The Open Access Publisher

For peer review only

Section/topic	#	Checklist item	Information reported Yes	No	Line number(s)
**Meta-bias(es)**	16	Specify any planned assessment of meta-bias(es) (e.g., publication bias across studies, selective reporting within studies)	☒	☐	286-287
**Confidence in cumulative evidence**	17	Describe how the strength of the body of evidence will be assessed (e.g., GRADE)	☒	☐	283-292

For peer review only

BioMed Central
The Open Access Publisher

Characteristics and practices of school-based cluster randomised controlled trials for improving health outcomes in pupils in the UK: a systematic review protocol

Journal:	*BMJ Open*
Manuscript ID	bmjopen-2020-044143.R1
Article Type:	Protocol
Date Submitted by the Author:	07-Dec-2020
Complete List of Authors:	Parker, Kitty; University of Exeter, NIHR ARC South West Peninsula<br>Nunns, Michael; University of Exeter, College of Medicine and Health<br>Xiao, ZhiMin; University of Exeter, Graduate School of Education<br>Ford, Tamsin; University of Cambridge,<br>Ukoumunne, Obioha; University of Exeter, NIHR ARC South West Peninsula (PenARC)
**Primary Subject Heading**:	Research methods
Secondary Subject Heading:	Health services research
Keywords:	STATISTICS & RESEARCH METHODS, Community child health < PAEDIATRICS, PUBLIC HEALTH

SCHOLARONE™
Manuscripts

I, the Submitting Author has the right to grant and does grant on behalf of all authors of the Work (as defined in the below author licence), an exclusive licence and/or a non-exclusive licence for contributions from authors who are: i) UK Crown employees; ii) where BMJ has agreed a CC-BY licence shall apply, and/or iii) in accordance with the terms applicable for US Federal Government officers or employees acting as part of their official duties; on a worldwide, perpetual, irrevocable, royalty-free basis to BMJ Publishing Group Ltd ("BMJ") its licensees and where the relevant Journal is co-owned by BMJ to the co-owners of the Journal, to publish the Work in this journal and any other BMJ products and to exploit all rights, as set out in our licence.

The Submitting Author accepts and understands that any supply made under these terms is made by BMJ to the Submitting Author unless you are acting as an employee on behalf of your employer or a postgraduate student of an affiliated institution which is paying any applicable article publishing charge ("APC") for Open Access articles. Where the Submitting Author wishes to make the Work available on an Open Access basis (and intends to pay the relevant APC), the terms of reuse of such Open Access shall be governed by a Creative Commons licence – details of these licences and which Creative Commons licence will apply to this Work are set out in our licence referred to above.

Other than as permitted in any relevant BMJ Author's Self Archiving Policies, I confirm this Work has not been accepted for publication elsewhere, is not being considered for publication elsewhere and does not duplicate material already published. I confirm all authors consent to publication of this Work and authorise the granting of this licence.

For peer review only

Characteristics and practices of school-based cluster randomised controlled trials for improving health outcomes in pupils in the UK: a systematic review protocol

Corresponding author

Ms Kitty Parker, NIHR ARC South West Peninsula, University of Exeter, Exeter, United Kingdom. Room 2.16, South Cloisters, St Luke's Campus, 79 Heavitree Rd, Exeter, EX1 1TX Email: kp477@exeter.ac.uk Telephone: (+44) 01392 727588

Co-authors

Dr Michael Nunns, College of Medicine and Health, University of Exeter, Exeter, United Kingdom.

Dr ZhiMin Xiao, Graduate School of Education, University of Exeter, Exeter, United Kingdom.

Professor Tamsin Ford, Department of Psychiatry, University of Cambridge, Cambridge, United Kingdom.

Dr Obioha C Ukoumunne, NIHR ARC South West Peninsula, University of Exeter, Exeter, United Kingdom

Word count: 3554, excluding title page, abstract, references, figures and tables

Key words

Cluster Randomised Trials

Schools

Systematic review

For peer review only

For peer review only - http://bmjopen.bmj.com/site/about/guidelines.xhtml

ABSTRACT

Introduction:

Cluster randomised trials (CRTs) are studies in which groups (clusters) of participants rather than the individuals themselves are randomised to trial arms. CRTs are becoming increasingly relevant for evaluating interventions delivered in school settings for improving the health of children. Schools are a convenient setting for health interventions targeted at children and the CRT design respects the clustered structure in schools (i.e., pupils within classrooms/teachers within schools). Some of the methodological challenges of CRTs, such as ethical considerations for enrolment of children into trials and how best to handle the analysis of data from participants (pupils) that change clusters (schools), may be more salient for the school setting. A better understanding of the characteristics and methodological considerations of school-based CRTs of health interventions would inform the design of future similar studies. To our knowledge this is the only systematic review to focus specifically on the characteristics and methodological practices of CRTs delivered in schools to evaluate interventions for improving health outcomes in pupils in the UK.

Methods and analysis:

We will search for CRTs published from inception to 30th June 2020 inclusively indexed in MEDLINE (Ovid). We will identify relevant articles through title and abstract screening, and subsequent full-text screening for eligibility against pre-defined inclusion criteria. Disagreements will be resolved through discussion. Two independent reviewers will extract data for each study using a pre-piloted data extraction form. Findings will be summarised using descriptive statistics and graphs.

Ethics and dissemination:

This methodological systematic review does not require ethical approval as only secondary data extracted from papers will be analysed and the data are not linked to individual participants. After completion of the systematic review, the data will be analysed, and the findings disseminated through peer-reviewed publications and scientific meetings. The review is registered on PROSPERO (Registration number: CRD42020201792).

For peer review only

For peer review only - http://bmjopen.bmj.com/site/about/guidelines.xhtml

ARTICLE SUMMARY

Strengths and limitations of this study

- To our knowledge, this is the first systematic review to describe the characteristics and methodological practices of school-based cluster randomised trials (CRTs) of health interventions in the UK.
- The review has a defined search strategy that is tailored to identifying school-based CRTs, eligibility criteria, and pre-piloted screening and data extraction strategies to minimise inaccuracies.
- Two independent reviewers will perform screening and data extraction, with any uncertainty resolved by consulting a third reviewer.
- The review will focus on studies conducted in the UK in order to align with available resources and create a relevant and focused review.
- There is the possibility that we are missing the opportunity to learn from studies in countries that have a similar education system to the UK.

For peer review only

INTRODUCTION

Cluster randomised trials (CRTs), also known as group randomised or place randomised trials, are studies in which groups (clusters) of participants (e.g., general practices, organisations, areas, etc) are randomly allocated to the trial arms, rather than the individual participants on whom outcomes are measured.[1] These studies are in contrast to the more traditional individual randomised trials, where the participants themselves are randomised. The CRT design is commonly used in health care research when interventions must be delivered at the cluster level and to minimise contamination of the trial arms that might otherwise occur when individuals are randomised.[2]

A characteristic feature of cluster randomised trials is that observations on participants who are in the same cluster are usually more similar than observations on participants who are from different clusters.[2] For example, patients registered with the same general practice are more likely to have similar health outcomes than those registered with different practices.[3] This similarity, or lack of statistical independence, between observations from the same cluster means that the usual procedures for calculating sample size and analysing data in individually randomised trials should not be used in CRTs.[1] The use of standard sample size methods are likely to result in studies that lack power to detect the specified intervention effect and the use of standard analytical methods may produce results that exaggerate evidence for the true effect of the intervention. Therefore, alternative methods should be used when conducting CRTs.

The intra-cluster correlation coefficient (ICC), denoted ρ, quantifies the similarity of observations of individuals within the same cluster. The ICC is the proportion of the total variability in the trial outcome that is between clusters (σ_b^2) as opposed to between individuals within clusters (σ_w^2):[4]

$$\rho = \frac{\sigma_b^2}{\sigma_b^2 + \sigma_w^2}$$

ρ can take values between 0 and 1. The larger ρ is, the greater the similarity between individuals within clusters, or equivalently the greater variability between clusters.

Information about ρ for the primary outcome is invaluable when designing a cluster randomised trial. It can be estimated from previous studies or feasibility studies, with a similar cluster structure and outcome to the planned trial.[5 6] Authors of cluster randomised trials should report estimates of their ICCs, ideally with confidence intervals because they

For peer review only - http://bmjopen.bmj.com/site/about/guidelines.xhtml

are usually based on studies with relatively few clusters,[7] to aid the design of future similar studies.

When calculating the sample size in CRTs one needs to determine the total number of clusters that need to be recruited and the number of individuals that need to be recruited from within each cluster. Methods for calculating the required sample size need to take the ICC into account. When the number of participants in each cluster is fixed and known in advance, the total number of individuals required in a CRT is calculated by inflating the number of individuals that would be required in an individual randomised trial by the *design effect* (*DE*), which is function of ρ:

$$DE = 1 + (n - 1)\rho$$

where n is the number of participants providing outcome data in each cluster (cluster size).[8] Having calculated the total number of participants required, this is divided by the cluster size to obtain the total number of clusters that is required. For scenarios where the total number of clusters available for a trial is known, an alternative calculation based on the same approach is used to calculate the number of participants that need to be recruited from each cluster.[3]

When estimating the intervention effect in CRTs, analytical methods that take account of ρ should be used,[1] otherwise confidence intervals will be too narrow and p values will be too small, resulting in an exaggeration of the amount of evidence for a true intervention effect.[9] Furthermore, the degrees of freedom used for calculating the confidence interval and p-value for the intervention effect should take account of the number of clusters.[10 11] A distinction can be made between statistical analyses that are carried out at the cluster level and those that are carried out at the individual level.[8] For cluster-level analyses the outcome is summarised for each cluster, for example by calculating the mean for continuous outcomes or percentages for binary outcomes across individuals in the cluster. Standard analytical methods are then used to compare the outcome between the trial arms using the cluster-level summary statistics as the observations. This method of analysis is valid because the cluster is both the unit of randomisation and the unit of analysis.[2] Alternatively, analyses of individual-level data involve the application of statistical methods that allow for the within cluster correlation.[2] This approach is exemplified by methods such as mixed effects (“multilevel”) models and marginal models estimated using Generalized Estimating Equations (GEEs).

For peer review only - http://bmjopen.bmj.com/site/about/guidelines.xhtml

CRTs are increasingly used to evaluate interventions for improving health outcomes in children.[12 13] Because of the amount of time children spend in school, it provides a natural setting in which interventions for preventing health problems can be delivered, participating children can be recruited, and health outcomes measured.[13] At a policy level there is increasing awareness of the potential for using the school setting to deliver, non-pharmacological, complex, prevention public health interventions.[12 14 15] Cluster randomisation is a more natural approach than individual randomisation in the school-based setting. It is often difficult to randomise individuals as pupils belong to predefined clusters (e.g., class, year group, school), and contamination between the trial arms can result as pupils interact within clusters. The CRT design respects the clustered structure in schools.

School-based cluster randomised trials share the same challenges of trials where other types of cluster are randomised. Within-cluster correlation is expected in school-based cluster randomised trials for a number of reasons: parents choose the schools their children attend and this may be related to factors associated with pupil outcomes; the school environment and culture will have a common influence on the pupils; pupils interact within schools and this can result in similar behaviours and outcomes.[12 13 16] Other recognised challenges may be even more salient for trials that randomise schools. There are additional ethical considerations for enrolment of children into trials to ensure pupils remain protected as research subjects.[12] Consent needs to be sought from several key agents including parents, pupils, head teachers and teachers. Consent for the school to be allocated the intervention is usually provided by the head teacher, but there may be interventions delivered to entire classes that some parents do not want their children to receive (e.g., aspects of sex education programmes that are not part of the standard curriculum). Retention of recruited pupils is an issue for trials that have a long follow-up duration and there is the need to consider how best to handle the analysis of data from pupils that change schools (clusters) during the course of the study.[17]

Several books have been published regarding CRT methodology.[1 2 18-20] In addition there have been a number of reviews of the conduct and reporting quality of cluster randomised trials.[12 21-26] One systematic review examined the characteristics and quality of reporting of CRTs worldwide involving children[12] and highlighted the specific difficulties of conducting such studies; nearly three quarters of the included studies randomised schools as the clusters. That review and our initial scoping research suggests a sharp increase in the number of these studies. No systematic review has specifically focussed on the characteristics of CRTs of health interventions delivered in the school-based setting in the UK. Such a review would: provide a pool of relevant knowledge for researchers planning

For peer review only - http://bmjopen.bmj.com/site/about/guidelines.xhtml

future similar trials in the UK; highlight good practices and common methodological challenges; obtain useful trial-based data on the intra-cluster (intra-school) correlation coefficient; provide relevant parameter values for simulation-based studies that use synthetic data to assess the statistical properties of methods used to analyse data from school-based cluster randomised trials.

This review aims to summarise the characteristics of, and methodological practices in, school-based CRTs with pupil health outcomes in the United Kingdom. The review examines several areas, including: participant characteristics; intervention type; recruitment, sampling and allocation methods; consent and ethical approval procedures; retention and analysis methods. The main outcome is a description of the methodological characteristics of school-based cluster randomised trials in the UK with a health outcome. Knowledge of the study characteristics and practices of researchers will greatly aid the design of cluster randomised trials in the school health setting.

METHODS

The systematic review will describe the characteristics of cluster randomised trials with health outcomes in the school setting. This section contains a description of the methodological strategy based on guidelines from the Preferred Reporting Items for Systematic Review and Meta-Analysis (PRISMA) statement.[27] The review is registered on PROSPERO (Registration number: CRD42020201792), an international register for systematic reviews, and any changes to the protocol will be recorded.

Search strategy

Peer reviewed articles written in English, published from inception to 30th June 2020 inclusively indexed in MEDLINE (Ovid) will be the source of data for this systematic review. The search strategy was developed following an initial scoping of the focus area and in consultation with an information specialist. The search strategy combined free text and index terms for the concepts *study type* (CRTs) and *schools* (Table 1). The study type concept was developed based on a sensitive MEDLINE search strategy for identification of cluster randomised trials developed by Taljaard et al.[28] Cluster design-related terms, ‘cluster*’, ‘group*’ and ‘communit*’ were combined with the terms ‘random’ and ‘trial’, along with ‘Schools’. The search was then

For peer review only - http://bmjopen.bmj.com/site/about/guidelines.xhtml

limited to English language as our resources make it unfeasible to translate papers (Table S1 in Supplementary Files).

Table 1: Search strategy implemented in this systematic review

Search strategy
Terms for Randomised Controlled trials:
1. random:.mp.
2. trial. ab,kw, ti.
Cluster design-related terms:
3. “cluster*”.ab, kw, ti.
4. “group*”.ab, kw, ti.
5. “communit*”.ab, kw, ti.
6. 3 OR 4 OR 5
School MESH term:
7. exp Schools/
Highest precision:
8. 1 AND 2 AND 6 AND 7
9. 8 limited to English language

Eligibility criteria

Eligible papers will be those reporting the results from school-based cluster randomised trials of health-related interventions in the UK for which there is a primary health outcome (including physical and mental health outcomes, health attitudes and well-being) measured on pupils.

The review will include participants who are children of school age in education in the UK. Participants are pupils in pre-school, primary school and secondary school settings. The types of eligible clusters include schools themselves, year groups, classrooms, teachers or any other relevant school-related unit. Any health-related intervention(s) will be considered. There must be a control/usual care comparison group within the published article. The primary outcome must be health related and measured on pupils.

All types of cluster randomised trial design are eligible, including parallel group, crossover and stepped wedge trials. Only definitive cluster randomised trials will be included. Only studies published in English will be included and we anticipate that all studies carried out in the UK will be published in English. If more than one publication of the primary outcome result for an eligible CRT is identified, a key study report (index paper) will be designated and used for data extraction.

For peer review only - http://bmjopen.bmj.com/site/about/guidelines.xhtml

Papers which do not report the main findings (primary outcome) will be excluded along with feasibility/pilot studies, protocol/design articles, process evaluations, economic evaluations/cost-effectiveness studies, statistical analysis plans, commentaries and papers reporting only findings from mediation/mechanism analyses. Studies for which the primary outcome is not health based (e.g., education attainment) will be excluded.

Screening and selection

All potentially eligible studies will undergo a two-stage screening process.

Stage 1: The titles and abstracts of the studies will be retrieved from MEDLINE (Ovid) and downloaded into Endnote (X9).[29] Any duplicate citations will be removed and remaining citations will be dual screened (KP & OU) for eligibility against the inclusion criteria above. Disagreements will be resolved through discussion.

Stage 2: Full text articles will be obtained for all papers that are potentially eligible following title and abstract screening. The reviewers (KP & OU) will evaluate articles based on the inclusion criteria using a pre-piloted coding method. Any discrepancies which cannot be resolved through discussion will be sent to a third reviewer (ZMX) for a decision.

Reviewers will keep a record of any studies excluded at each step. Results will be reported using a PRISMA flow diagram.

Risk of Bias (quality) assessment

A risk of bias assessment is not necessary for this methodological review as we are not interested in the specific estimates of intervention effect in the included studies. We seek only to describe characteristics of the studies. Some of the information extracted from the papers is indicative of quality in cluster randomised trials and this will be summarised as part of our review.

Data extraction

A data extraction form will be piloted on a random sample of 10 included papers. Any modifications to the form will be made following the pilot. KP will extract data from all eligible papers, while OU will check extraction. Any uncertainty will be resolved by consulting a third reviewer (ZMX). Information will be recorded using a data extraction form in Microsoft Excel.

For peer review only - http://bmjopen.bmj.com/site/about/guidelines.xhtml

The following data will be extracted from included articles: characteristics of the participating schools and pupils; intervention type and mode of delivery; health condition/aspect targeted by the intervention; justification for using cluster trial design; unit of randomisation (i.e., type of cluster); school-level (or other cluster-level) characteristics used to balance the randomisation; allocation ratio; length of follow-up; number of follow-ups; target sample size (i.e., number of schools and pupils); assumptions underlying sample size (e.g., intra-cluster correlation coefficient, loss to follow-up); committee that provided ethical approval; activities covered by the consent agreements; primary outcome; reporter of primary outcome (e.g., teacher, parent, pupil); method of data collection; achieved sample size; number of schools (clusters) and pupils that were lost to follow-up; analysis method used to estimate intervention effect; baseline factors that were adjusted for in the analysis; value of the intra-cluster correlation coefficient in the primary analysis model; methodological challenges that were highlighted by the authors.

Missing information that is not available in the included papers will be obtained from corresponding protocol papers and other sibling publications for the studies. Authors may be contacted for missing or incomplete information and given 2 weeks to respond.

Data analysis

No formal sample size in terms of the number of required eligible papers has been calculated because we are seeking to obtain all published school-based cluster randomised trials in the United Kingdom to date published in MEDLINE (Ovid). Meta-analysis is not appropriate as the review is focused on summarising methodological characteristics. Study characteristics will be summarised using means and standard deviations (or median and interquartile ranges) for continuous variables, and numbers and percentages for categorical variables. Appropriate graphs (e.g., histograms, line graphs, scatterplots) will also be used to summarise specific features of the data. Challenges reported by authors will be summarised narratively.[30] Statistical analysis will be performed using Stata 16.[31]

Patient and Public Involvement

There has been no contribution from patients or the public to the design of this systematic review protocol.

For peer review only - http://bmjopen.bmj.com/site/about/guidelines.xhtml

DISCUSSION

To our knowledge, this is the first systematic review to describe the characteristics and methodological practices of school-based cluster randomised trials of health interventions in the UK. We have a defined search strategy that is tailored to identifying school-based CRTs, selection criteria and a pre-piloted extraction strategy. Pilot testing, and subsequent screening and data extraction will be conducted by two independent reviewers, with disagreements resolved by consulting a third reviewer. In doing this we hope to minimise inaccuracy. Additionally, the review aims to cover a range of CRTs conducted in schools for a variety of different health conditions/areas.

Identifying CRTs is challenging as many papers do not explicitly use the word 'cluster' in the title or abstract. We have included terms in our search such as 'group' randomised and 'community' randomised to try and improve the sensitivity, thus widening the search so not to miss any eligible papers. We have also used the exploded MESH term, 'exp School/', in the hope of identifying publications that may state schools or classes as their unit of randomisation.

This review will summarise data using descriptive statistics. Meta-analysis is not used here to pool ICC estimates. Our initial scoping of the literature indicated that most papers do not report the standard error of the ICC, which is required for pooling the estimates. Furthermore, the studies to be included in the review will be methodologically and clinically diverse (e.g., different outcomes and health conditions). There is, therefore, no true single underlying ICC; rather there is a range of true ICCs specific to different scenarios. A single pooled ICC from a meta-analysis would not be meaningful and would obscure nuances about how its size depends on the context of the study. It is more useful to summarise the variability in the estimated ICCs as this provides a range of values within which to assess the sensitivity of the sample size calculation to uncertainty about the true value of the ICC in a given scenario.[32]

We have conducted extensive scoping searches in order to best identify the studies of interest. A limitation of the review is that we will limit our search to the MEDLINE (Ovid) database, thus potentially missing out on articles published in other journals (e.g. mental health interventions published in Psych Info). MEDLINE was used because our research question is to describe the characteristics of trials that evaluate the impact of health interventions on health outcomes. The database includes journals of interest for both physical health and mental health. We have also chosen not to examine grey literature therefore potentially missing out on studies with greater methodological challenges. Also there will be no forward and backward citation searching, but we do have a clearly defined

For peer review only - http://bmjopen.bmj.com/site/about/guidelines.xhtml

population of papers. Feasibility studies have been excluded, but there are different learning issues from such studies that will be the subject of a separate review. These decisions have been taken to enable the review to be more focused and time-effective.

The review is focused on health-based CRTs in schools. There is a wider literature of other types of intervention (particularly in educational research) that have been evaluated in this setting using the CRT design, but, given the limited resources and the large number of potentially eligible studies identified during scoping, it was considered more relevant and efficient to restrict the review to studies in the health area.[33]

Another limitation of this review is the difficulty in identifying CRTs as many papers do not explicitly use the word 'cluster' in the title or abstract. Therefore, we have included terms in our search such as 'group' and 'community' to try and improve the sensitivity, thus widening the search so not to miss any eligible papers. We have also used the exploded MESH term, 'exp School/', in the hope of identifying publications that may state schools or classes as their unit of randomisation.

A pragmatic decision has been made to focus on UK studies in order to align with available resources and create a relevant and focused review. There is the possibility that we are missing out on the opportunity to learn from studies in countries that have a similar education system to the UK. Our scoping searches established that there is a considerably large number of eligible papers and we restricted the study eligibility to the UK. Ideally, we would include papers globally, but this is not practical. Despite being focussed on the UK, the findings of this review will be of wider interest as many methodological challenges in the design of cluster randomised trials will be similar across some countries.

Because of the amount of time children spend in school, it provides a natural setting in which interventions for preventing health problems and improving health outcomes in children can be delivered and evaluated.[13] Cluster randomised controlled trials in the school-based setting are particularly relevant for non-pharmacological interventions, such as social programmes aimed at improving public health[13] and the use of this study design is increasing.[12] Through summarising the methodological aspects of health-related cluster randomised controlled trials conducted in a schools, this review will provide methodology-related knowledge specific to these trials which will help researchers plan future similar studies effectively in the UK and elsewhere.

For peer review only - http://bmjopen.bmj.com/site/about/guidelines.xhtml

Ethics and dissemination: This methodological systematic review does not require ethical approval as only secondary data extracted from papers will be analysed and the data are not linked to individual participants. After completion of the systematic review, the data will be analysed, and the findings disseminated through peer-reviewed publications and scientific meetings.

Data statement: This is a systematic review of published studies. No additional data will be available beyond that which will be included in the report of the systematic review findings.

Author contributions: KP and OU conceptualised the study. KP drafted the manuscript and incorporated comments from authors for successive drafts. MN, ZMX and TF contributed to the design and content. All authors read and approved the final manuscript. KP is the guarantor of the review.

Funding statement: This work was supported by the National Institute for Health Research (NIHR) Applied Research Collaboration South West Peninsula (NIHR ARC South West Peninsula). Our funded group National Institute for Health Research Applied Research Collaboration South West Peninsula does not have a grant number because we are part of the NIHR infrastructure. The views expressed are those of the author(s) and not necessarily those of the NHS, the NIHR or the Department of Health and Social Care.

Acknowledgments: Kitty Parker was supported by a PhD studentship funded by the National Institute for Health Research Applied Research Collaboration South West Peninsula (NIHR ARC South West Peninsula). Obioha Ukoumunne was supported by the NIHR ARC South West Peninsula. The views expressed are those of the author(s) and not necessarily those of the NHS, the NIHR or the Department of Health and Social Care.

Competing interests statement. None declared.

For peer review only

References

1. Eldridge S, Kerry S. A Practical Guide to Cluster Randomised Trials in Health Services Research. Chichester, West Sussex: John Wiley & Sons 2012.
2. Donner A, Klar N. Design and Analysis of Cluster Randomization Trials in Health Research: Wiley 2000.
3. Campbell MJ. Cluster randomized trials in general (family) practice research. *Stat Methods Med Res* 2000;9(2):81-94. doi: 10.1177/096228020000900202 [published Online First: 2000/08/18]
4. Eldridge S, Ukoumunne O, Carlin J. The intra-cluster correlation coefficient in cluster randomized trials: a review of definitions. *International Statistical Review* 2009;77:378 - 94.
5. Kerry SM, Bland JM. The intracluster correlation coefficient in cluster randomisation. *BMJ* 1998;316(7142):1455. doi: 10.1136/bmj.316.7142.1455 [published Online First: 1998/06/06]
6. Eldridge SM, Ukoumunne OC, Carlin JB. The Intra-Cluster Correlation Coefficient in Cluster Randomized Trials: A Review of Definitions. *International Statistical Review* 2009;77(3):378-94. doi: 10.1111/j.1751-5823.2009.00092.x
7. Ukoumunne OC. A comparison of confidence interval methods for the intraclass correlation coefficient in cluster randomized trials. *Stat Med* 2002;21(24):3757-74. doi: 10.1002/sim.1330 [published Online First: 2002/12/17]
8. Eldridge S, Kerry S. A Practical Guide to Cluster Randomised Trials in Health Services Research: Wiley 2012.
9. Bland JM, Kerry SM. Statistics notes. Trials randomised in clusters. *BMJ* 1997;315(7108):600. doi: 10.1136/bmj.315.7108.600 [published Online First: 1997/09/26]
10. Kahan BC, Forbes G, Ali Y, et al. Increased risk of type I errors in cluster randomised trials with small or medium numbers of clusters: a review, reanalysis, and simulation study. *Trials* 2016;17(1):438. doi: 10.1186/s13063-016-1571-2
11. Cornfield J. Randomization by group: a formal analysis. *Am J Epidemiol* 1978;108(2):100-2. doi: 10.1093/oxfordjournals.aje.a112592 [published Online First: 1978/08/01]
12. Walleser S, Hill SR, Bero LA. Characteristics and quality of reporting of cluster randomized trials in children: reporting needs improvement. *Journal of Clinical Epidemiology* 2011;64(12):1331-40. doi: https://doi.org/10.1016/j.jclinepi.2011.04.006
13. Goesling B. A Practical Guide to Cluster Randomized Trials in School Health Research. *Journal of School Health* 2019;89(11):916-25. doi: 10.1111/josh.12826
14. Crocetti MT, Amin DD, Scherer R. Assessment of risk of bias among pediatric randomized controlled trials. *Pediatrics* 2010;126(2):298-305. doi: 10.1542/peds.2009-3121 [published Online First: 2010/07/14]
15. Thomson D, Hartling L, Cohen E, et al. Controlled trials in children: quantity, methodological quality and descriptive characteristics of pediatric controlled trials published 1948-2006. *PLoS One* 2010;5(9) doi: 10.1371/journal.pone.0013106 [published Online First: 2010/10/12]
16. Shackleton N, Hale D, Bonell C, et al. Intraclass correlation values for adolescent health outcomes in secondary schools in 21 European countries. *SSM Popul Health* 2016;2:217-25. doi: 10.1016/j.ssmph.2016.03.005
17. Schweig JD, Pane JF, McCaffrey DF. Switching cluster membership in cluster randomized control trials: Implications for design and analysis. *Psychological Methods* 2020;25(4):516-34. doi: 10.1037/met0000258
18. Campbell M, Walters S. How to Design, Analyse and Report Cluster Randomised Trials in Medicine and Health Related Research. Hoboken, NJ: John Wiley & Sons 2014.
19. Hayes R, Moulton L. Cluster Randomised Trials. Boca Raton, FL: Chapman and Hall/CRC Press 2009.
20. Murray D. Design and Analysis of Group-Randomized Trials. New York: Oxford University Press 1998.

For peer review only - http://bmjopen.bmj.com/site/about/guidelines.xhtml

21. Eldridge SM, Ashby D, Feder GS, et al. Lessons for cluster randomized trials in the twenty-first century: a systematic review of trials in primary care. *Clin Trials* 2004;1(1):80-90. doi: 10.1191/1740774504cn006rr [published Online First: 2005/11/12]
22. Fiero MH, Huang S, Oren E, et al. Statistical analysis and handling of missing data in cluster randomized trials: a systematic review. *Trials* 2016;17:72-72. doi: 10.1186/s13063-016-1201-z
23. Ivers NM, Taljaard M, Dixon S, et al. Impact of CONSORT extension for cluster randomised trials on quality of reporting and study methodology: review of random sample of 300 trials, 2000-8. *BMJ* 2011;343:d5886. doi: 10.1136/bmj.d5886
24. Donner A, Brown KS, Brasher P. A Methodological Review of Non-Therapeutic Intervention Trials Employing Cluster Randomization, 1979–1989. *Int J Epidemiol* 1990;19(4):795-800. doi: 10.1093/ije/19.4.795
25. Simpson JM, Klar N, Donnor A. Accounting for cluster randomization: a review of primary prevention trials, 1990 through 1993. *Am J Public Health* 1995;85(10):1378-83. doi: 10.2105/ajph.85.10.1378 [published Online First: 1995/10/01]
26. Varnell SP, Murray DM, Janega JB, et al. Design and analysis of group-randomized trials: a review of recent practices. *Am J Public Health* 2004;94(3):393-9. doi: 10.2105/ajph.94.3.393 [published Online First: 2004/03/05]
27. Moher D, Liberati A, Tetzlaff J, et al. Preferred reporting items for systematic reviews and meta-analyses: the PRISMA statement. *BMJ* 2009;339:b2535. doi: 10.1136/bmj.b2535
28. Taljaard M, McGowan J, Grimshaw JM, et al. Electronic search strategies to identify reports of cluster randomized trials in MEDLINE: low precision will improve with adherence to reporting standards. *BMC Med Res Methodol* 2010;10:15-15. doi: 10.1186/1471-2288-10-15
29. EndNote [program]. EndNote X9 version. Philadelphia, PA: Clarivate, 2013.
30. Popay J, Roberts H, Sowden A, et al. Guidance on the conduct of narrative synthesis in systematic reviews: A product from the ESRC Methods Programme2006.
31. Stata16 [program]. Release 16 version. College Station, TX: StataCorp LLC, 2019.
32. Turner RM, Thompson SG, Spiegelhalter DJ. Prior distributions for the intracluster correlation coefficient, based on multiple previous estimates, and their application in cluster randomized trials. *Clinical Trials* 2005;2(2):108-18. doi: 10.1191/1740774505cn072oa
33. Torgerson D. Designing randomised trials in health, education and the social sciences: an introduction: Springer 2008.

For peer review only

For peer review only - http://bmjopen.bmj.com/site/about/guidelines.xhtml

Table S1: MEDLINE search strategy

MEDLINE Search strategy
Terms for Randomised Controlled trials:
1. random:.mp.
2. trial. ab,kw, ti.
Cluster design-related terms:
3. “cluster*”.ab, kw, ti.
4. “group*”.ab, kw, ti.
5. “communit*”.ab, kw, ti.
6. 3 OR 4 OR 5
School MESH term:
7. exp Schools/
Highest precision:
8. 1 AND 2 AND 6 AND 7
9. 8 limited to English language

For peer review only

PRISMA-P 2015 Checklist

This checklist has been adapted for use with systematic review protocol submissions to BioMed Central journals from Table 3 in Moher D et al: Preferred reporting items for systematic review and meta-analysis protocols (PRISMA-P) 2015 statement. *Systematic Reviews* 2015 **4**:1

An Editorial from the Editors-in-Chief of *Systematic Reviews* details why this checklist was adapted - **Moher D, Stewart L & Shekelle P:** Implementing PRISMA-P: recommendations for prospective authors. *Systematic Reviews* 2016 **5**:15

Section/topic	#	Checklist item	Information reported		Line number(s)
			Yes	No	
**ADMINISTRATIVE INFORMATION**					
**Title**					
Identification	1a	Identify the report as a protocol of a systematic review	☒	☐	3
Update	1b	If the protocol is for an update of a previous systematic review, identify as such	☐	☒	N/A
**Registration**	2	If registered, provide the name of the registry (e.g., PROSPERO) and registration number in the Abstract	☒	☐	55
**Authors**					
Contact	3a	Provide name, institutional affiliation, and e-mail address of all protocol authors; provide physical mailing address of corresponding author	☒	☐	6-21
Contributions	3b	Describe contributions of protocol authors and identify the guarantor of the review	☒	☐	378-381
**Amendments**	4	If the protocol represents an amendment of a previously completed or published protocol, identify as such and list changes; otherwise, state plan for documenting important protocol amendments	☐	☒	N/A
**Support**					
Sources	5a	Indicate sources of financial or other support for the review	☒	☐	383-388
Sponsor	5b	Provide name for the review funder and/or sponsor	☒	☐	383-388
Role of sponsor/funder	5c	Describe roles of funder(s), sponsor(s), and/or institution(s), if any, in developing the protocol	☒	☐	390-394
**INTRODUCTION**					
**Rationale**	6	Describe the rationale for the review in the context of what is already known	☒	☐	172-185

For peer review only

For peer review only - http://bmjopen.bmj.com/site/about/guidelines.xhtml

Section/topic	#	Checklist item	Information reported Yes	Information reported No	Line number(s)
**Objectives**	7	Provide an explicit statement of the question(s) the review will address with reference to participants, interventions, comparators, and outcomes (PICO)	☒	☐	187-194
**METHODS**					
**Eligibility criteria**	8	Specify the study characteristics (e.g., PICO, study design, setting, time frame) and report characteristics (e.g., years considered, language, publication status) to be used as criteria for eligibility for the review	☒	☐	218-239
**Information sources**	9	Describe all intended information sources (e.g., electronic databases, contact with study authors, trial registers, or other grey literature sources) with planned dates of coverage	☒	☐	206-207
**Search strategy**	10	Present draft of search strategy to be used for at least one electronic database, including planned limits, such that it could be repeated	☒	☐	206-216
STUDY RECORDS					
Data management	11a	Describe the mechanism(s) that will be used to manage records and data throughout the review	☒	☐	243-244, 264-265
Selection process	11b	State the process that will be used for selecting studies (e.g., two independent reviewers) through each phase of the review (i.e., screening, eligibility, and inclusion in meta-analysis)	☒	☐	241 – 252
Data collection process	11c	Describe planned method of extracting data from reports (e.g., piloting forms, done independently, in duplicate), any processes for obtaining and confirming data from investigators	☒	☐	261-265
**Data items**	12	List and define all variables for which data will be sought (e.g., PICO items, funding sources), any pre-planned data assumptions and simplifications	☒	☐	267-279
**Outcomes and prioritization**	13	List and define all outcomes for which data will be sought, including prioritization of main and additional outcomes, with rationale	☒	☐	187-194, 267-279
**Risk of bias in individual studies**	14	Describe anticipated methods for assessing risk of bias of individual studies, including whether this will be done at the outcome or study level, or both; state how this information will be used in data synthesis	☒	☐	254-259
DATA					
**Synthesis**	15a	Describe criteria under which study data will be quantitatively synthesized	☒	☐	284-293
	15b	If data are appropriate for quantitative synthesis, describe planned summary measures, methods of handling data, and methods of combining data from studies, including any planned exploration of consistency (e.g., I^2, Kendall's tau)	☒	☐	284-293
	15c	Describe any proposed additional analyses (e.g., sensitivity or subgroup analyses, meta-regression)	☒	☐	284-293
	15d	If quantitative synthesis is not appropriate, describe the type of summary planned	☒	☐	284-293

BioMed Central The Open Access Publisher

Section/topic	#	Checklist item	Information reported		Line number(s)
			Yes	No	
**Meta-bias(es)**	16	Specify any planned assessment of meta-bias(es) (e.g., publication bias across studies, selective reporting within studies)	☒	☐	287-288
**Confidence in cumulative evidence**	17	Describe how the strength of the body of evidence will be assessed (e.g., GRADE)	☒	☐	284-293

For peer review only

BioMed Central
The Open Access Publisher
